# Deciphering the molecular basis of invasiveness in *Sdhb*-deficient cells

**DOI:** 10.18632/oncotarget.5106

**Published:** 2015-10-08

**Authors:** Céline Loriot, Mélanie Domingues, Adeline Berger, Mélanie Menara, Maëva Ruel, Aurélie Morin, Luis-Jaime Castro-Vega, Éric Letouzé, Cosimo Martinelli, Alexis-Pierre Bemelmans, Lionel Larue, Anne-Paule Gimenez-Roqueplo, Judith Favier

**Affiliations:** ^1^ INSERM, UMR970, Paris Cardiovascular Research Centre, F-75015 Paris, France; ^2^ Université Paris Descartes, Sorbonne Paris Cité, Faculté de Médecine, F-75006 Paris, France; ^3^ INSERM, U1021, CNRS UMR3347, Institut Curie, F-91405 Orsay, France; ^4^ INSERM, U968, Institut de la vision, F-75012 Paris, France; ^5^ Université Pierre et Marie Curie Paris 06, F-75005 Paris, France; ^6^ Programme Cartes d'Identité des Tumeurs, Ligue Nationale Contre Le Cancer, F-75013 Paris, France; ^7^ CEA, DSV, I^2^BM, Molecular Imaging Research Center (MIRCen), F-92265 Fontenay-aux-Roses, France; ^8^ CNRS, CEA URA 2210, F-92265 Fontenay-aux-Roses, France; ^9^ Assistance Publique-Hôpitaux de Paris, Hôpital Européen Georges Pompidou, Service de Génétique, F-75015 Paris, France; ^10^ Rare Adrenal Cancer Network-Cortico Médullosurrénale Tumeurs Endocrines, Institut National du Cancer, F-75014 Paris, France

**Keywords:** paraganglioma, SDHB, metastasis, EMT, keratin 19

## Abstract

Metastatic pheochromocytomas and paragangliomas (PPGL) are malignant neuroendocrine tumors frequently associated with germline mutations in the *SDHB* gene. *SDHB*-mutated PPGL display a hypermethylator phenotype associated with hallmarks of epithelial-to-mesenchymal transition (EMT). In the present study, we report the characterization of a unique model of *Sdhb* knockout in mouse chromaffin cells. *Sdhb* deficient cells exhibit a metastatic phenotype as highlighted by increased individual cell migration (characterized by faster motility and increased persistence) as well as high invasive and adhesion abilities. This phenotype is associated with the modulation of *Twist1, Twist2, Tcf3, Snai1, N-cadherin* or *Krt19* expression, reflecting an EMT-like reprogramming of cells. *Krt19* is epigenetically silenced in *Sdhb*-deficient cells and re-expressed after treatment by the demethylating agent *decitabine*. *Krt19* rescue by lentiviral transduction in *Sdhb*-deficient cells and *Krt19* inhibition by RNA interference in wild-type cells were performed. Both studies revealed the involvement of KRT19 in the invasive phenotype by modulating collective and individual migration and cell/extra-cellular matrix adhesion properties. These findings underline the role of hypermethylation and EMT in the *in vitro* acquisition of metastatic properties, following SDHB loss of function.

## INTRODUCTION

Pheochromocytomas and paragangliomas (PPGL) are rare neuroendocrine tumors that arise from chromaffin cells of the adrenal medulla and from sympathetic or parasympathetic ganglia, respectively. In about 40% of cases, PPGL are inherited, which implies that a germline mutation has been identified in one of the 13 known PPGL predisposition genes: the *RET* and *HIF2A* proto-oncogenes and the *NF1*, *VHL*, *SDHx* (*SDHA*, *SDHB*, *SDHC*, *SDHD* and *SDHAF2*), *MAX*, *TMEM127*, *FH* and *MDH2* tumor suppressor genes [1, 2]. Somatic mutations in *NF1*, *VHL*, *MAX*, *HIF2A* and *HRAS* genes have also been identified in around 30% of cases [3].

Among PPGL susceptibility genes, *SDHB* is specifically associated with malignancy and poor prognosis. Patients carrying an *SDHB* mutation are more likely to develop a metastatic form of their disease with a median survival substantially reduced, compared to patients without *SDHB* mutation [4]. Additionally, time lapse between diagnosis and development of metastasis is shorter for *SDHB* mutation carriers than others (for whom it can last up to 20 years). However, it remains unclear why *SDHB*-mutation carriers are predisposed to these aggressive forms of the disease and genomic studies have failed in identifying a causative genomic event, concomitant to SDHB loss, that would explain this peculiar phenotype [5, 6].

We previously proposed epithelial-to-mesenchymal transition (EMT) as the process likely to be responsible for *SDHB*-related malignancy in PPGL. Using transcriptome profiling of a large cohort of PPGL, we reported that *SDHB*-malignant samples displayed an overexpression of *LOXL2*, *TWIST1*, *TCF3*, *MMP1*, *MMP2* and a down regulation of *CDH2* and *KRT19*, which are all hallmarks of the EMT process. Furthermore, the nuclear retention of SNAIL was specifically observed in *SDHB*-malignant tumors, suggesting an active form of this key EMT transcription factor in this subgroup of aggressive tumors [7].

EMT is a physiological embryonic process that allows, among others, neural crest cells migration after neural tube closure. It can also be found in pathological conditions such as fibrosis [8] and metastatic dissemination [9]. Cancer cells, which are submitted to EMT undergo a myriad of morpho-physiological and epigenetic changes, including but not limited to the loss of cell junctions, increased proteolytic abilities and enhanced migratory and invasive capacities [10]. During malignant progression, tumor cells lose cell/cell and cell/extracellular matrix adhesion properties, which can be assessed by decreased actin-dependent focal adhesion or keratins-dependent hemi desmosomes [11]. Keratins (KRT, also called cytokeratins) are part of intermediate filaments. Among the 54 keratins [12], keratin 19 (KRT19) is the smallest one, and is of peculiar interest because of its implication as a diagnostic biomarker of circulating cells in breast cancer [13]. Loss or down regulation of KRT19 is associated with an aggressive behavior characterized by enhanced migratory, adhesive and invasive properties in squamous cell carcinomas, neuroblastomas, renal and breast cancers [14–19]. *KRT19* extinction is due to the methylation of its promoter, as described in uterine leiomyoma, renal cell carcinoma, as well as in neuroblastoma, another neural crest cell derived tumor [17, 20, 21]. This was described to be associated with high-grade tumors, and thus with reduced patients’ survival [15, 16].

We and others, recently demonstrated that mutations in *SDHx* genes (encoding succinate dehydrogenase subunits) lead to succinate accumulation, which inhibits DNA demethylases (TET enzymes), leading to a global hypermethylation of DNA [22–25]. In *SDHB*-mutated PPGL and in *Sdhb−/−* immortalized mouse chromaffin cells (imCC), we demonstrated that *KRT19* was one of the most hypermethylated and down-regulated gene [24].

Following these observations, we evaluated here the consequences of SDHB loss and the role of KRT19 in the establishment of a metastatic phenotype following *in vitro* inactivation of *Sdhb* in murine chromaffin cells.

## RESULTS

### *Sdhb−/−* immortalized mouse chromaffin cells display an EMT-like metastatic phenotype

**Figure 1:**
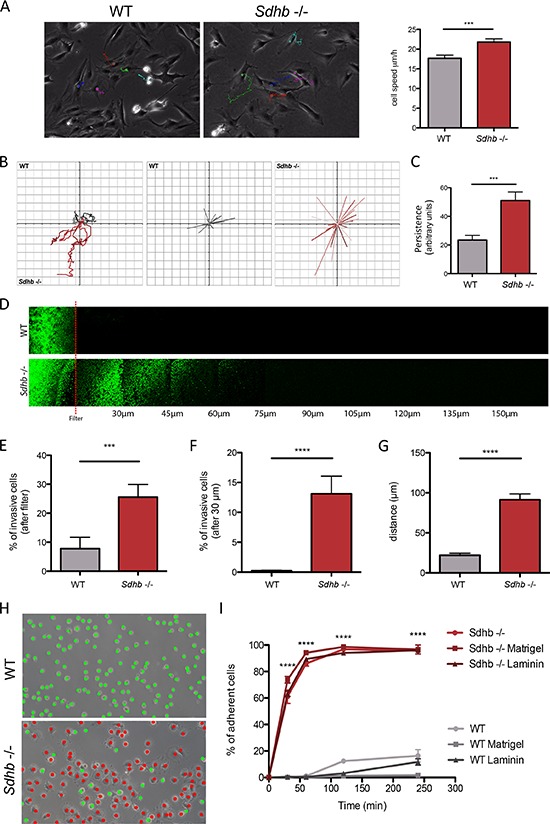
Characterization of changes in migratory and invasive properties following *Sdhb* loss **A.** Individual cells speed was estimated by tracking cell nucleus during 12 hours. ***p = 0.0003. **B.** Vector displacement diagrams representing total distance (left panel), and persistence (middle and right panel). **C.** Comparison of persistence. ***p = 0.007. **D.** Cells invasion's abilities were evaluated through their capacity to move into a Matrigel matrix. Red line symbolized the filter. **E.** Quantification of cell abilities to pass through the filter. ***p = 0.004. **F.** Quantification of cell invasion, cells were considered invasive beyond 30 μm. ****p < 0.0001. **G.** Mean distance travelled by cells into Matrigel matrix. ****p < 0.0001. **H.** Cell adhesion abilities were measured by counting adherent (marked as red dots) versus non-adherent (marked as green dots) cells one hour after plating. Scale bar 125 μm. ****p < 0.0001. **I.** Adhesion course: quantification of cell adhesion during 240 minutes. ****p < 0.0001 *See also Supplementary Movies 1 and 2*.

We have previously reported the initial characterization of the *Sdhb−/−* imCC cell line, which displays a hypermethylator phenotype associated with increased collective migration capacities and a reduced proliferation [24]. Observation of cell morphology revealed a mesenchymal aspect of *Sdhb−/−* cells, reminiscent of an EMT phenotype (Supplementary Figure 1). Individual cell migration assessed by single cell tracking analysis revealed that *Sdhb−/−* imCC migrate at a mean speed of 22 μm/h, which is significantly faster than control cells (17 μm/h) (Figure 1A and Supplementary Movies 1 and 2). Consistently, as a result of enhanced individual cell migration, total distance was significantly higher in *Sdhb−/−* imCC compared to wild-type (WT) cells. Interestingly, vector displacement diagrams revealed that in contrast with WT cells which tend to go round in circles, *Sdhb*-deficient cells aim to move away from their point of origin and follow a linear path (Figure 1B), further increasing the travelled distance or persistence (Figure 1C).

We next performed inverted invasion assays in a Matrigel^®^ matrix, to determine whether *Sdhb−/−* imCC are able to actively pass through an 8 μm filter, digest Matrigel^®^ matrix, and migrate beyond a 30 μm arbitrary limit, classically used for such experiments (Figure 1D). Very few WT cells were able to get through the filter, while 25% of *Sdhb−/−* imCC did (Figure 1E). Accordingly, about 60% of the latter were then able to migrate beyond the arbitrary 30 μm limit; while none of the WT cells did (Figure 1F). Interestingly, *Sdhb−/−* imCC were found up to 180 μm after the filter, with a 90 μm mean distance, compared to 20 μm for WT cells (Figure 1G), showing real skills to progress into the matrix.

The ability of passing through the filter, digesting extracellular matrix, coupled with high migration rates mimic the different steps that occur during the process of intravasation (or trans-endothelial migration). We then looked at cell-substratum adhesion, without coating, or on plate coated with Matrigel^®^ or laminin. We observed that cell adhesion is equivalent whatever the conditions used, and that one hour after plating, approximately 90% of *Sdhb−/−* imCC are adherent whereas WT cells hardly adhere during the same period (Figures 1H and 1I). Three hours after plating, only about 20% of WT cells were adherent.

### The EMT-like phenotype is associated with transcriptional reprogramming of *Sdhb−/−* immortalized mouse chromaffin cells

**Figure 2:**
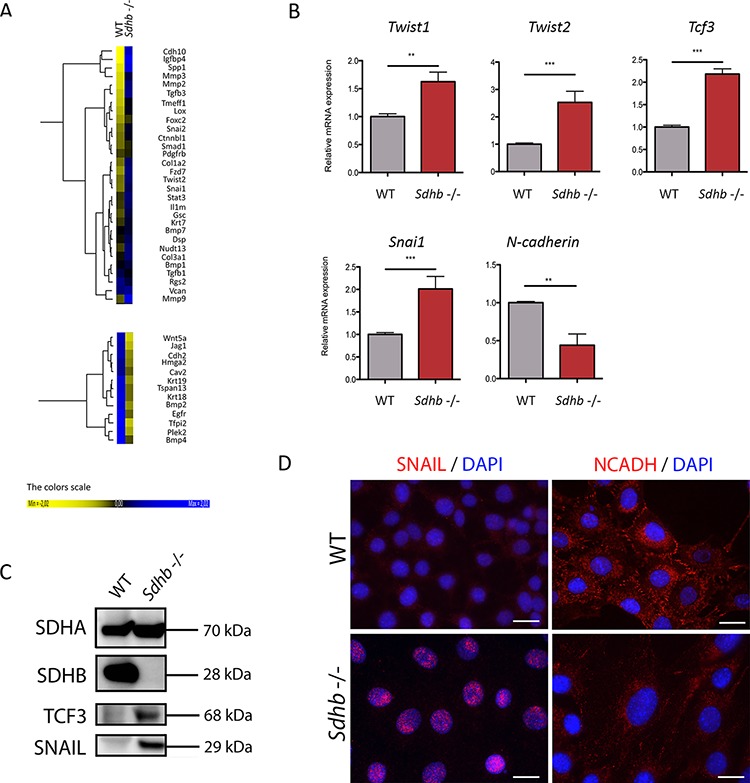
Molecular characterization of the EMT process in *Sdhb*−/− imCC **A.** Heat map samples of the most differentially expressed EMT associated genes between WT and *Sdhb−/−* imCC. **B.** qRT-PCR analyses showing *Twist1*, *Twist2*, *Tcf3*, and *Snai1* inductions as well as and *N-cadherin* loss of expression in *Sdhb* deficient imCC compared to WT cells. **p = 0.0079 and *p* = 0.0099; ***p = 0.0002. Data are shown as fold change relative to WT **C.** SNAIL and TCF3 were studied at protein level using western blotting and specific antibodies. **D.** SNAIL and N-Cadherin were studied using immunofluorescence and specific antibodies. Scale bar 20 μm *See also Supplementary Figures 2 and 3*

**Figure 3:**
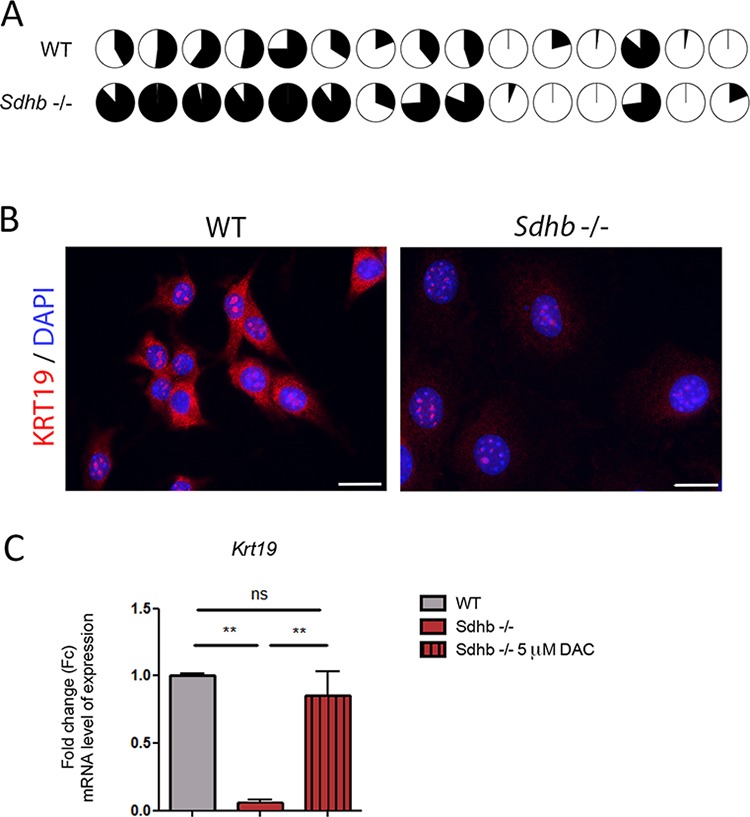
KRT19 loss of expression is driven by hypermethylation **A.** Differential methylation rate of *Krt19* promoter in *Sdhb*-deficient cells compared to WT cells. Fifteen CpG islands were studied in *Krt19's* promoter. Filled circles figured percentage of methylation of CpG islands. **B.** KRT19 was studied using immunofluorescence and specific antibody. Labeling was seen is the cytosol together with a non-specific nucleolar staining. Scale bar 20 μm. **C.** qRT-PCR analyses showing *Krt19* loss of expression in *Sdhb*-deficient cells compared to WT cells, which is reversed after *decitabine* treatment. **p = 0.0075 and *p* = 0.0025. Data are shown as fold change relative to WT *See also Supplementary Figures 4 and 5*.

Transcriptomic analysis of genes associated with EMT was performed to compare WT and *Sdhb−/−* imCC and revealed variations concordant with an activation of EMT in *Sdhb*-deficient cells (Figure 2A, and Supplementary Figure 2). Using qRT-PCR, we validated that the expression of the main markers of EMT, *Twist1*, *Twist2*, *Tcf3* and *Snai1* were significantly increased, while *N-cadherin* and *Krt19*'s expression were significantly decreased in *Sdhb−/−* imCC (Figure 2B and 3C). The overexpression of TCF3 and SNAIL proteins was further demonstrated by western blot in *Sdhb*-deficient cells (Figure 2C) and immunofluorescence analysis showed the nuclear localization of SNAIL as well as down-regulation of N-cadherin and KRT19 proteins in *Sdhb−/−* imCC (Figure 2D and 3B).

Extravasation requires cells to roll and to adhere to endothelial cells, both process involving adhesion molecules such as integrins, galectins, focal adhesion kinases (FAK), selectins or mucins [26]. Interestingly, transcriptome analyses showed a significant overexpression of *Cd24a*, *Muc1*, *Lgals3*, *Lgals3bp*, *Lgals9*, *Ptk2* and integrins α1, α8, α11, β3 and β5 mRNAs in *Sdhb−/−* imCC compared to WT cells (Supplementary Figure 3), underscoring the high adherent properties of *Sdhb*-deficient cells. Moreover trans-endothelial migration, which requires the loss of adherent junctions between endothelial cells, or their retraction, can be promoted by β-catenin and VEGF [26]. Interestingly, we observed that both *Ctnnb1* and *Vegfa* mRNA were overexpressed in *Sdhb−/−* imCC (Supplementary Figure 3). Altogether, these data established that loss of SDHB promotes a transcriptional reprogramming that is associated with an EMT phenotype in the mouse chromaffin cell line.

### *Krt19* is hypermethylated and downregulated in *Sdhb−/−* imCC

In human PPGL, transcriptome and methylome data revealed that the levels of *KRT19* mRNA or its promoter's methylation were able to distinguish *SDHB*-mutated tumors from all others tumors (Supplementary Figures 4A and 4B). We thus decided to further investigate KRT19 functions in *Sdhb−/−* imCC. We observed that *KRT19* hypermethylation did occur in the murine cell line following *Sdhb* gene inactivation as pointed out by comparing the methylation of 15 CpG islands in *Krt19* promoter by representative reduced bisulfate sequencing (Figure 3A). There was a global 30% increase in DNA methylation at the *Krt19* locus in *Sdhb−/−* imCC compared to WT cells (Supplementary Figure 5). This was associated with the absence of KRT19 protein in *Sdhb−/−* imCC (Figure 3B) as a result of a 10-fold decrease in gene expression evaluated by quantitative RT-PCR (Figure 3C). *Decitabine* treatment led to the re-expression of *Krt19* in *Sdhb*-deficient cells to levels comparable to that observed in WT cells, thus demonstrating that the marked reduction of *Krt19* expression was indeed the result of increased methylation (Figure 3D).

### KRT19 rescue partially reverses *Sdhb−/−* imCC metastatic hallmarks

**Figure 4:**
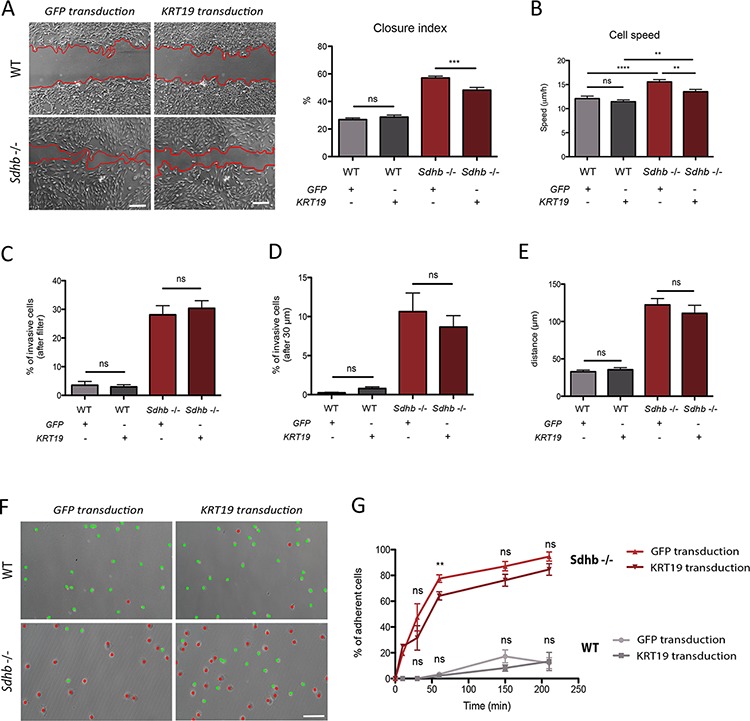
KRT19 rescue after lentiviral transduction and its functional consequences **A.** Collective migration was followed using a wound scratch assay. Cells were studied for migration during 10 hours. Migration is represented as the closure percentage of the wound. Scale bar 250 μm. ***p = 0.0002; ns, non-significant. **B.** Individual cells speed was estimated by tracking cell nucleus during 12 hours. **p = 0.0013; ns, non-significant. **C.** Quantification of cell abilities to pass through the filter. ns, non-significant. **D.** Quantification of cell invasion, cells were considered invasive beyond 30 μm. ns, non-significant. **E.** Mean distance travelled by cells into Matrigel matrix. ns, non-significant. **F.** Cell adhesion abilities were measured by counting adherent (marked with red dots) versus non-adherent (marked with green dots) cells one hour after plating. Scale bar 125 μm. **G.** Adhesion course: quantification of cell adhesion during 210 minutes. **p = 0.0013; ns, non-significant *See also Supplementary Figures 6 and 7*.

To evaluate the participation of KRT19 to the metastatic phenotype of *Sdhb*-deficient cells, we infected cells with a lentivirus encoding the complete *Krt19* cDNA. A GFP-expressing lentivirus was used as a control. Although KRT19 lentiviral transduction induced a massive up-regulation of *Krt19* mRNA level in both cell types (1,000-fold induction, Supplementary Figure 6A), immunofluorescence analysis showed no differences on protein staining levels detected in WT cells while in *Sdhb−/−* imCC, it allowed the detection of the protein at a staining level comparable to that observed in WT cells (Supplementary Figure 6B). We evaluated the impact of KRT19 rescue on the metastatic traits previously described. KRT19 rescue led to a significant (but not complete) inhibition of collective migration with a closure index modestly but significantly decreased from 60% to 48% in *Sdhb−/−* imCC and with no effect on WT cells (26% in GFP-transduced cells compared to 28% in KRT19-transduced cells) (Figure 4A).

Single cell tracking experiments showed that KRT19 rescue induced a partial but significant reduction of *Sdhb−/−* imCC speed (from 16 μm/h to 14 μm/h), while it had no effect on WT cells (12 μm/h in both conditions) (Figure 4B). Vector displacement diagrams did not reveal any difference in cells’ movement after protein rescue and persistence remained unchanged (Supplementary Figure 7A and 7B). KRT19 rescue had no effect on the invasive capacities of *Sdhb−/−* imCC (Figure 4C–4E) and only mildly delayed their adherence. After 1 h, 64% of cells had adhered as compared to 78% with GFP transduction (Figures 4F and 4G) but ultimately, the same percentage of cells adhered at the end of the time-lapse studied (Figure 4G).

### KRT19 transient inhibition in WT cells enhances cell's adhesion

**Figure 5:**
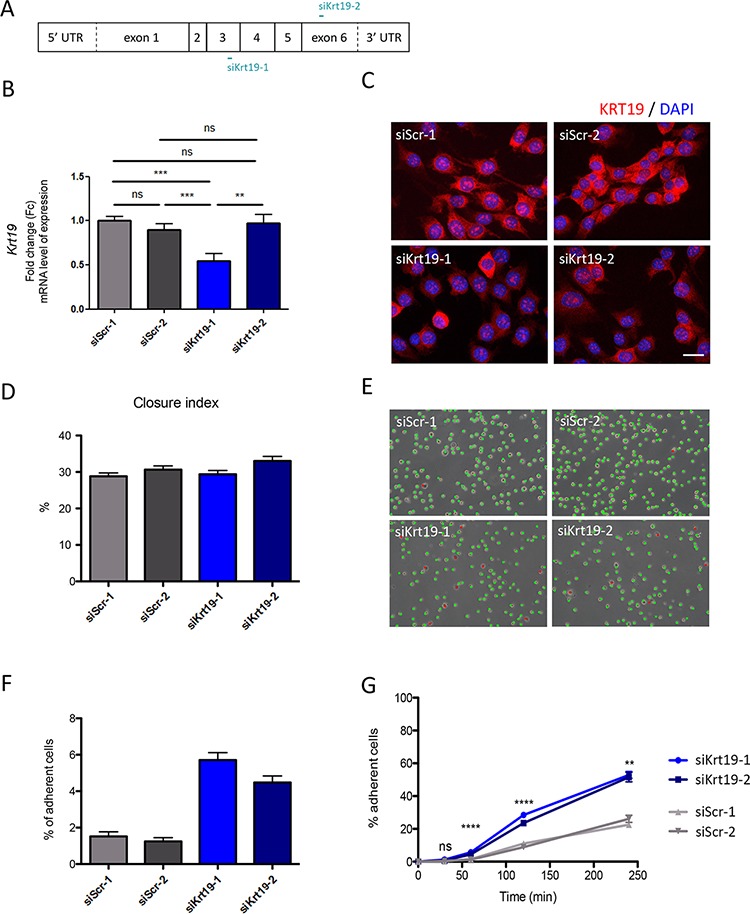
KRT19 transient inhibition by siRNA and its functional consequences **A.** Graphical representations of *Krt19* mRNA and localization of siRNA interaction. **B.** KRT19 inhibition was confirmed in WT cells mRNA level using qRT-PCR. As a control cells were independently transfected with the scrambles siRNA. **p = 0.0030; ***p = 0.0003 and *p* = 0.0007; ns, non-significant. Data are shown as fold change relative to siScr-1 **C.** KRT19 inhibition was studied cells at protein level using immunofluorescence. Cells were independently transfected with either one of the scrambles siRNA (siScr-1 or siScr-2) as a control (upper panels), or with one of Krt19 directed siRNA (siKrt19-1 and siKrt19-2) (bottom panels). Scale bar 20 μm. **D.** Collective migration was followed using a wound scratch assay. Cells were studied for migration during 10 hours. Migration is represented as the closure percentage of the wound. **E.** Cell adhesion abilities were measured by counting adherent (marked with red dots) versus non-adherent (marked with green dots) cells one hour after plating. Scale bar 125 μm. **F.** Quantification of cell adhesion one hour after plating. **G.** Adhesion course: quantification of cell adhesion during 240 minutes. **p = 0.0014; ****p < 0.0001; ns, non-significant

*Krt19* expression was inhibited in WT cells using two different siRNAs sequence (Figure 5A). A significant reduction in *KRT19* mRNA levels was achieved with only one: si_KRT19_1 (Figure 5B), while both led to a marked decrease in protein levels in a large proportion of transfected cells (Figure 5C), suggesting either transcriptional or translational repression mechanisms.

KRT19 inhibition in WT cells had no effect on global cell migration, as evaluated by the wound scratch assays (Figure 5D). In contrast, it induced a significant increase in cell adherence, one hour after plating (Figure 5E and 5F), which reached 53% of all cells 4 hours after plating (*vs* 24% with scramble siRNAs) (Figure 5G).

## DISCUSSION

In the present study, we demonstrate that *Sdhb* inactivation alone is sufficient to promote the *in vitro* transformation of mouse chromaffin cells into invasive cells. We show that *Sdhb* knockout leads to a molecular reprogramming compatible with the activation of an EMT-like process, as highlighted by both transcriptional and protein modulations of *Twist1/2*, *Tcf3*, *Snai1*, *N-cadherin* and *Krt19*, previously shown to be differentially expressed in *SDHB*-malignant human PPGL [7]. Our data support the functional role of the epigenetically silenced *Krt19* gene, whose repression participates to some of the phenotypic modifications observed in *Sdhb*-deficient cells. Altogether, these data establish that *Sdhb* does act as a tumor suppressor gene by promoting epithelial-to-mesenchymal transition and that KRT19 is one actor of this process.

Succinate dehydrogenase (SDH) is composed of four subunits (SDHA-D), each encoded by a different nuclear gene. While mutations in the 4 *SDHx* genes lead to a complete loss of SDH activity and predispose to PPGL, it is still unclear why *SDHB* inactivation only is responsible for metastatic forms of the disease. Two major hypotheses may explain this difference: i) the coexistence of another genetic or genomic alteration that would act in addition or in synergy with SDHB inactivation; or ii) another, yet unidentified, function of SDHB protein, that would mediate EMT in case of *SDHB* mutation. Our results in the *Sdhb* knockout cell model are in favor of the second one and suggest that *SDHB* inactivation is sufficient to induce both PPGL tumorigenesis and the EMT-associated metastatic phenotype.

Referring to ‘epithelial-to-mesenchymal transition’ in the context of PPGL can be debated, given the non-epithelial state of chromaffin cells. It would be more appropriate to use the term ‘neuroendocrine- to-mesenchymal transition’ (neuroendoMT). In particular, we did not observe the loss of E-cadherin, which is widely used as a hallmark of EMT in epithelial cells, as it is hardly expressed by WT imCC (data not shown). However, we observed the loss of N-Cadherin after *Sdhb* inactivation. Interestingly, a comparable process has been described in endothelial cells, which are submitted to an EMT-like phenomenon, occurring during fibrosis. This process, referred to as ‘endothelial-to-mesenchymal transition’ (endoMT) is associated with the loss of the only expressed cadherin: the VE-cadherin [8, 27].

In human tumors and in *Sdhb−/−* imCC, SDHB loss is responsible for succinate dehydrogenase inactivation, succinate accumulation, and subsequent inhibition of 2-oxoglutarate dependent dioxygenases [22, 24]. Of the dioxygenases described to date, TETs enzymes are responsible for DNA demethylation, and are inhibited by succinate, leading to global hypermethylation of more than 4000 genes, including *KRT19*. Intermediate filaments have been described as being implicated in cell migration and adhesion [11, 18] as well as in cell invasion [14, 18]. By being one of the most significantly hypermethylated, and one of the most significantly down-regulated gene, *KRT19* was a pertinent candidate to decipher the still misunderstood link between *SDHB* deficiency, hypermethylation, EMT and malignancy. This work identifies KRT19 as one of the important actors implicated in migratory and adhesive properties of *Sdhb−/−* imCC and thus in *SDHB* associated metastatic phenotype. However, *in vitro* results after KRT19 rescue in *Sdhb*-deficient cells show that KRT19 repression has only a very mild impact on invasion (despite a trend to a reduced ability to pass through the filter and of the percentage of invasive cells). Altogether, our data show that in addition to KRT19, other EMT actors are implicated in the establishment of the metastatic skills we observed. Further studies will be needed to investigate other target candidates and search for eventual synergic effects with KRT19.

With the validation of the first experimental cellular model that mimics the *SDHB*-related metastatic phenotype in chromaffin cells, this study opens a new research area for *SDHB*-related oncongenesis. We show that SDHB loss alone induces a neuroendoMT phenomenon associated with increased metastatic abilities and likely explaining the bad prognosis caused by *SDHB* mutations in patients. KRT19 is one of the partners implicated in the establishment of the mesenchymal phenotype and in the process that drives PPGL malignancy.

## MATERIALS AND METHODS

### Cell line

Wild-type *(Sdhb^lox/lox^)* immortalized mouse chromaffin cells (imCC) were isolated from the adrenal medulla of a genetically modified mouse harboring LoxP sites on both sides of exon 2 of the *Sdhb gene* [28]. After *spontaneous in vitro* immortalization, *Sdhb* knockout was performed using an adenovirus expressing Cre recombinase [24]. Cell morphology was assessed by lucent phase microscopy (Supplementary Figure 1A) and by immunostaining of their tubulin cytoskeleton (Supplementary Figure 1B).

### Cell culture

imCC were cultured in DMEM (Dulbecco Modified Eagle Medium, Gibco) with 4.5% FBS (Fetal bovine serum, Gibco) and 1% antibiotics (penicillin/streptomycin, Gibco). Cells were grown at 37°C, in 5% CO_2_. Experiments were performed using one of the two imCC clones: clone 8 and its WT counterpart [24]. Except when mentioned, all experiments were performed using this regular medium, on regular plastic plates and dishes, without coating.

### Single cell tracking

Exponentially growing cells were seeded at a density of 5 × 10^4^ in a six-well plate. After 24 h of incubation, the cells were imaged every 4 min for 12 h. The nucleus of each cell was manually tracked with the Manual Tracking plugin for *ImageJ*. Experiments were performed three times, with at least 25 cells tracked each time.

### Invasion assay

A Matrigel matrix was previously polymerized in a Transwell system (BD Biosciences); 5 × 10^4^ cells were seeded on in complete medium and left to adhere for 4 h. After 3 washes in medium without FBS, cells were allowed to invade the matrix during 5 days, at 37°C, with 5% CO_2_. Medium with FBS was added on top of the Matrigel matrix as a stimulus. After 1 h of calcein staining, living cells were followed using confocal microscopy. For each experiment, each sample was performed in duplicate and 3 areas of the Transwell were studied. Experiments were performed three times.

### Adhesion assay

After complete detachment from their plate using trypsin-EDTA 0.05% (Gibco), 5 × 10^4^ cells were plated in 6-wells dishes. Matrigel^®^ coating was performed with pure Matrigel^®^ (BD Biosciences), allowed to polymerized 30 minutes at 37°C. Laminin coating was performed using 100 μg/ml solution, allowed to polymerized 1 hour at room temperature. Cell adhesion was followed by taking picture during 3 h. Adherent and non-adherent cells were identified on the basis of their morphology (round and refractive cells were considered as non-adherent). Cells were marked on the image with a green dot if considered as non-adherent and with a red dot if considered as adherent and the respective numbers of dots were quantified. Experiments were performed three times.

### Human tumors methylome analysis

*KRT19* methylation data was a part of global DNA methylation study performed on a 145 samples PCC/PGL collection the Illumina Infinium HumanMethylation27 assay, as described elsewhere [24].

### Human tumors transcriptome analysis

*KRT19* level of expression data were a part of EMT study performed on 188 samples of PCC and PGL and using the HG-U133 Plus 2.0 Affymetrix Gene Chip arrays, as described elsewhere [7].

### imCC transcriptome analysis

Gene expression profiles of wild-type and *Sdhb−/−* imCC c8 were assessed in duplicates using the Gene Chip^®^ Mouse Gene 1.0 ST Array. Data are available as Array Express entry E-MTAB-3403.

### imCC methylome analysis

*Krt19* methylation data was a part of global DNA methylation study performed on imCC using Reduced Representation Bisulphite Sequencing technique (RRBS), as previously described [24].

### Decitabine treatment

A daily fresh prepared and filtered solution of decitabine (A3656, Sigma Aldrich) was used to treat cells, during 5 days. Drug (0.25 mg) was resuspended in DMSO and diluted in culture medium. A classical (5 μM) [29] dose was used; not-treated cells received medium with equivalent amount of DMSO only.

### Quantitative Real-Time (qRT)-PCR

Total RNAs were extracted from cell pellets using RNA Extract II kit^®^ (Macherey-Nagel), as described by the manufacturer. Three μg of RNA were treated with DNase Amp Grade and EDTA (Invitrogen) during 10 min at 65°C. Reverse transcription was performed on 1.5 μg of RNA, using random primers and Superscript Reverse transcriptase (Invitrogen), during 1 h at 42°C. RNA concentrations were determined with a NanoVue Plus (GE Healthcare ^®^) and subjected to qRT-PCR in triplicate, using PTC-200 DNA Engine^®^ *Thermal Cycler*. Normalization was performed with Ubc and 18S amplifications, and comparisons were calculated using the ΔΔCt method. Primers’ sequences were as follows:

*Ubc*: F 5′-AGCCCAGTGTTACCACCAAG-3′; R 5′-ACCCAAGAACAAGCACAAG-3′;

*18S*: F 5′-CGCGGTTCTATTTTGTTGGT-3′; R 5′-AACCATAAACGATGCCGAC-3′;

*Twist1*: F 5′-CTGGACTCCAAGATGGCAAGC TG-3′; R 5′-ACCTAGATGTCATTGTTTCCAGAGAA-3′

*Twist2*: F 5′-CATGTCCGCCTCCCACTA-3′; R 5′-ACCTTGTGGCTCCTCATGAC-3′

*Tcf3*: F 5′-TGGCACTTACAGTGGGACTTC-3′; R 5′-GTTGAGGGGCTAGGTGAGAA-3′

*Snai1*: F 5′-TGGAAAGGCCTTCTCTAGGC-3′; R 5′-CCACTGCAACCGTGCTTTT-3′

*Krt19*: F 5′-GGTGCCACCATTGACAACTC-3′; R 5′-GAGACAGAACACGCCTTGC-3′;

*Cdh2*: F5′-GAAGATGTTTACAGCGCAGTCTT-3′; R 5′-AAGTTCAGTATGAAAGCAGCGAG-3′

### Immunofluorescence assay

For immunofluorescence assays, cells plated on glass slides were washed in PBS and fixed for 10 min in ice-cold paraformaldehyde 4% or methanol 100%. Antigen retrieval was performed with Tris-EDTA buffer (1 mM EDTA pH = 8, 0.005% Twin 20; pH = 9) or with citrate buffer (0.01M sodium citrate, 0.05M citric acid, pH = 6), during 45 seconds in a microwave. After blockade of aspecific sites (1% BSA, 0.1% Triton), primary antibodies were incubated for 90 min at room temperature. The antibodies used were: anti-SDHA (Abcam, Ab14715), anti-SNAI1 (Abcam, Ab85931), anti-KRT19 (Thermo RB9021), anti-N-cadherin (Abcam, Ab12221), and anti α-tubulin (Sigma-Aldrich). Secondary antibodies, conjugated with Alexa Fluor 488 or with Alexa Fluor 594 (1/2000, Invitrogen), were incubated for 1 h, at room temperature in the dark. Slides were finally mounted in Vectashield, containing DAPI, for a blue nuclear staining. Acquisitions were performed using Axioimager Z1 Zeiss, with apotome system.

### Western blot analysis

Total proteins were extracted, in Tris-EDTA buffer (Tris NaCl pH = 7.4, 0.5% NP-40, 0.5% deoxycholic acid and 5 mM EDTA) containing proteases inhibitors cocktail. Protein concentrations were determined with Bradford colorimetric method. 50 μg were resolved on NuPAGE^®^ Novex 4–16% Bis-Tris gradient gel (Invitrogen), transferred on PVDF membrane. After blocking in PBS-milk solution (5%), membranes were incubated with specific primary antibodies, followed by incubation with corresponding HRP-conjugated secondary antibodies. Revelation was performed using ECL Plus (Pierce). The antibodies used were: anti-SDHA (Abcam, Ab14715), anti-SDHB (Sigma, HPA002868), anti-SNAI1 (Abcam, Ab85931), and anti-TCF3 (Abcam, Ab69999).

### Promoter methylation analysis

Genomic DNA was treated with bisulfite, using Epitect Bisulfite kit (Qiagen), following manufacturer's recommendations. Fifteen CpG island, of *Krt19's* promoter, were studied by pyrosequencing using Pyromark Q24 instrument and software, and following manufacturer's recommendations.

### Plasmid construction

GFP (Green Fluorescent Protein) and KRT19 (Keratin19) cDNA were inserted in a lentiviral shuttle plasmid by Gateway recombination (Life Technologies, Saint-Aubin, France) under the transcriptional control of the PGK (Phosphoglycerate Kinase) promoter. The recombinant lentiviral genome contained a self-inactivating deletion of the left LTR and the central polypurine tract/central termination sequence (cPPT/CTS) and was previously described [30].

### Virus production and cell transduction

Recombinant lentiviral particles were produced by transient transfection of HEK-293T cells as previously described [30]. Viral supernatants were concentrated by ultracentrifugation at 70,000 g for 90 min at 4°C. Finally, to achieve a 1,000-fold concentration of the initial supernatant, viral pellets were resuspended in a minimal volume of PBS. Aliquots of 5–10 μl were then stored at −80°C until further use. Total particle concentration of the viral stocks was estimated by quantification of the p24 capsid protein using RETRO-TEK HIV-1 p24 Antigen ELISA kit (ZeptoMetrix, Buffalo, New York, United States) according to the manufacturer's instructions. For transduction of 5 × 10^4^ cells, a multiplicity of transduction (MOI) of 2 ng of p24 protein/cell was used.

### Wound scratch assay

Cells were seeded at confluence in 6-well dishes. Twenty-four hours after plating, a wound was made using a 10 μL tip and cells were allowed to migrate for 10 h. Relative cell migration was established by calculating the closure index, based on the analysis of at least 25 pictures for each time point and condition, using *ImageJ* software, as described [24]. The experiments were performed in triplicates.

### siRNA transient transfection

For KRT19 inhibition in WT cells, custom siRNA were designed and tested. Two of them were chosen as they induced an important Krt19 inhibition, siKrt19-1: 5′ UGGCCUACCUGAAGAAGAA 3′ and siKrt19-2: 5′ GCCAGAACCAGGAGUAUAA 3′. siRNA were then transfected using HiPerfect transfection reagent (Qiagen), along with cell plating, and cells were collected 36 hours after transfection. Two different negative controls siRNA were used, as they did not react with any known mouse mRNA. Scrambles siRNA were design as follow, siScr-1: 5′ UACGCAUUCGUCAUUGCUA 3′ and siScr-2: 5′ UCGAAGUAUUCCGCGUACG 3′.

### Statistical analysis

All statistical analyses were performed using Graph Pad Prism software. Data are represented as mean (of at least 3 independent experiments) ± SEM (standard error of the mean).

## SUPPLEMENTARY FIGURES AND VIDEOS


