# Supplementary material for: Deciphering the molecular basis of invasiveness in Sdhb-deficient cells
Source: Oncotarget. 2015 Oct 8;6(32):32955–65. doi: 10.18632/oncotarget.5106 (PMC4741742; doi:10.18632/oncotarget.5106)
Supplement: Supplementary file 1 [file oncotarget-06-32955-s001.pdf]

SUPPLEMENTARY FIGURES AND VIDEOS

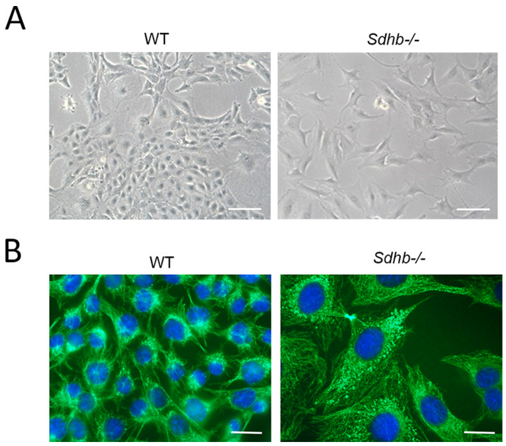

Supplementary Figure S1: Morphological characterization of Sdhb−/− imCC. A. Cell morphology observation by white light microscopy. Scale bar 125 µm. **B.** Cell morphology observed by β-tubulin staining using immunofluorescence. Scale bar 20 µm.

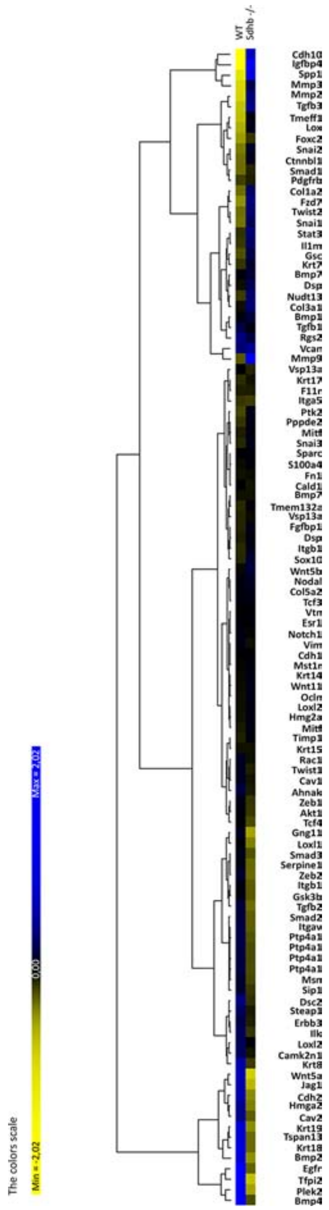

Supplementary Figure S2: Related to Figure 2: Complete heat map of EMT-associated transcriptome data in WT and Sdhb−/− imCC.

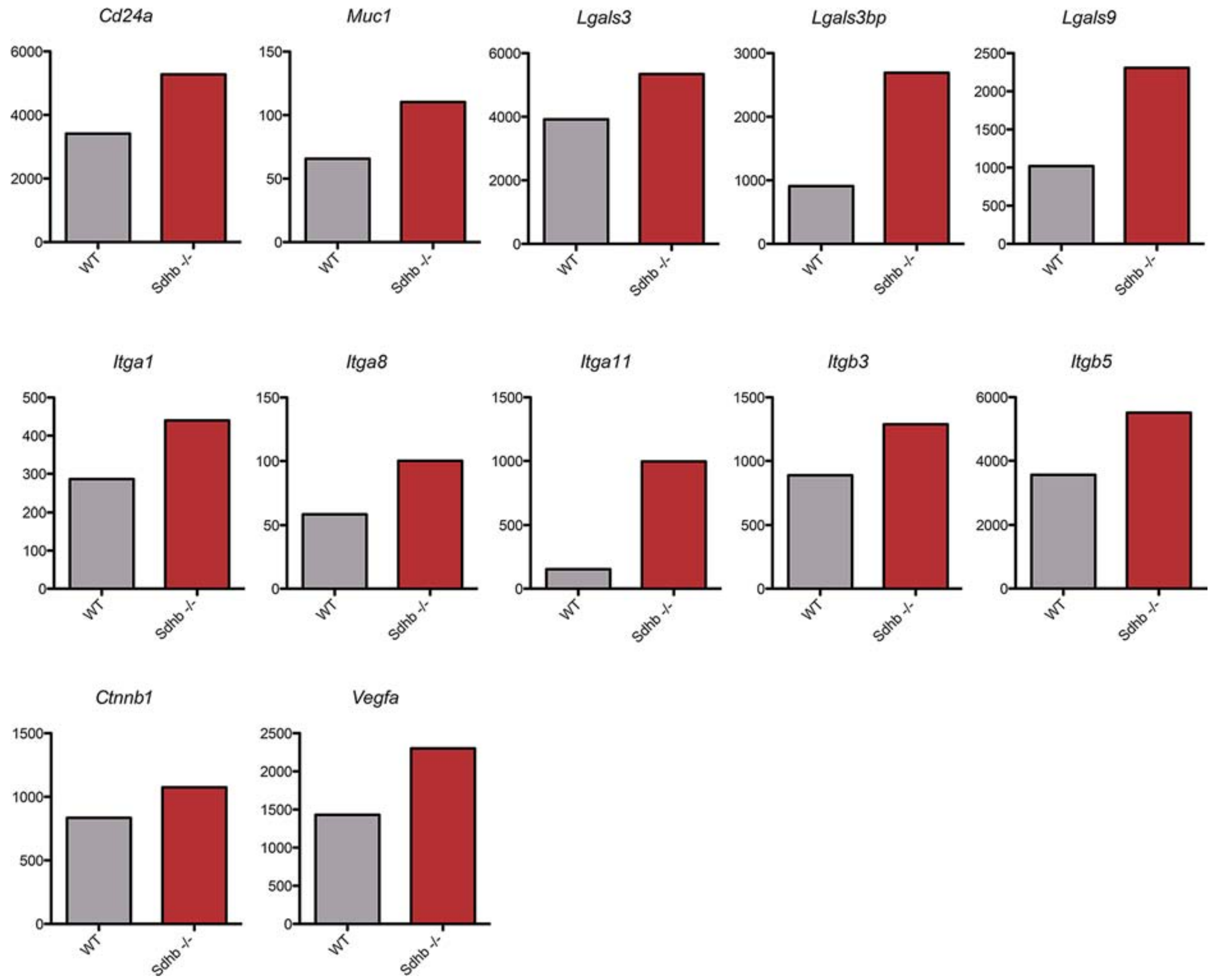

Supplementary Figure S3: Related to Figure 2: Transcriptomic data correlated with high adhesive skills observed in Sdhb−/− cells compared to WT cells.

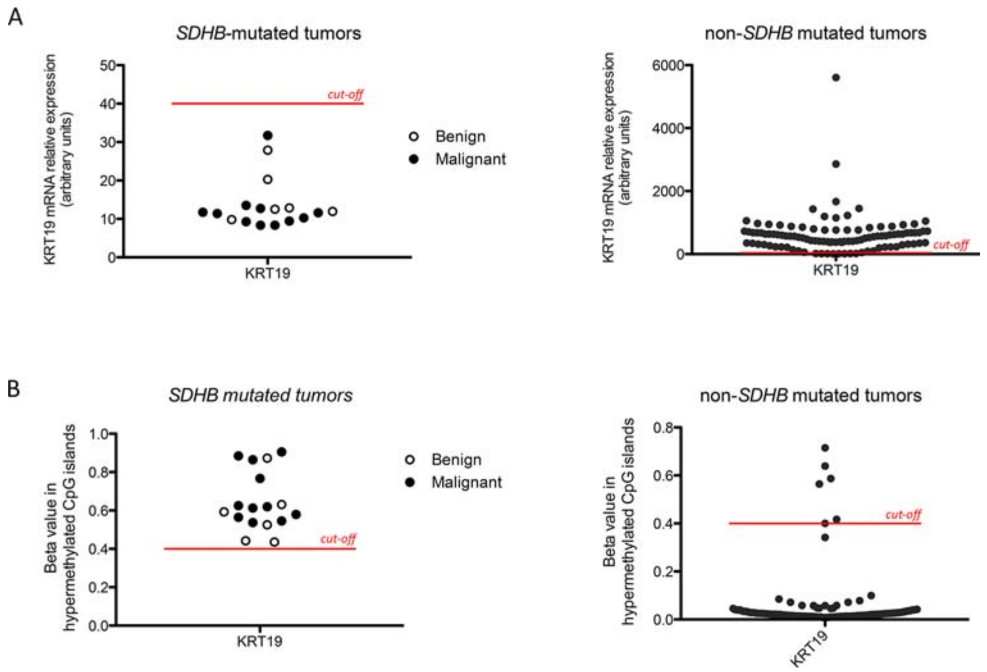

Supplementary Figure S4: Related to Figure 3: The choice of KRT19 as the best gene candidate. Cut-off allowing separating SDHB-mutated tumors from all others, based on KRT19 mRNA level of expression and promoter methylation. **A.** All SDHB-mutated tumors had a KRT19 expression level inferior to the defined cut-off, while non-SDHB mutated tumors had expression levels equivalent or superior to the cut-off **B.** All SDHB-mutated tumors had a KRT19 promoter methylation mean level superior to the defined cut-off, while all but 6 non-SDHB mutated tumors had KRT19 promoter methylation mean level inferior to the cut-off. Each dot represents a tumor sample and its relative level of KRT19 mRNA expression or KRT19 promoter methylation.

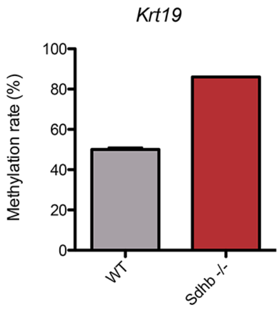

Supplementary Figure S5: Related to Figure 3: Krt19 promoter methylation in mouse chromaffin cells. Differential methylation rate of Krt19 promoter in Sdhb deficient cells compared to WT cells. Representation of imCC methylome data obtained using RRBS technique.

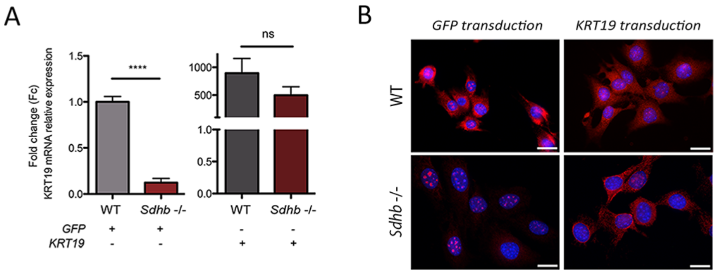

Supplementary Figure S6: Related to Figure 4: KRT19 efficient rescue in Sdhb−/− imCC. A. KRT19 transduction validation was performed at mRNA level using qRT-PCR. Cells were independently transduced with the same lentiviral particles encoding GFP, as a control. ****p < 0.0001; ns, non-significant. Data are shown as fold change relative to GFP-transduced WT cells **B.** KRT19 rescue was confirmed in Sdhb−/− imCC at protein level using immunofluorescence. As a control cells were independently transduced with the same lentiviral particles encoding GFP. Scale bar 20 µm.

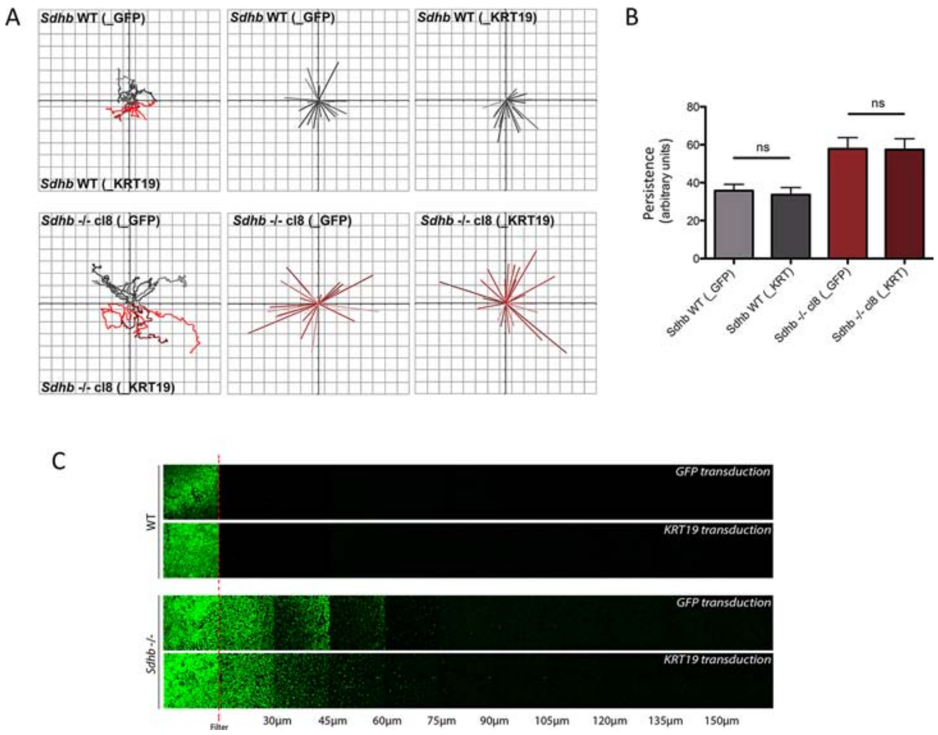

Supplementary Figure S7: Related to Figure 4: Persistence and invasion evaluation after KRT19 rescue. A. Vector diagrams representing total distance (left panels), and displacement (middle and right panels) of WT and Sdhb−/− imCC with or without KRT19 rescue **B.** Comparison of displacements after KRT19 rescue. Data are represented as mean ± SEM. ns, non-significant **C.** Involvement of KRT19 in cell invasion was assessed though inverted invasion assay in a Matrigel matrix. Red line symbolized the filter.

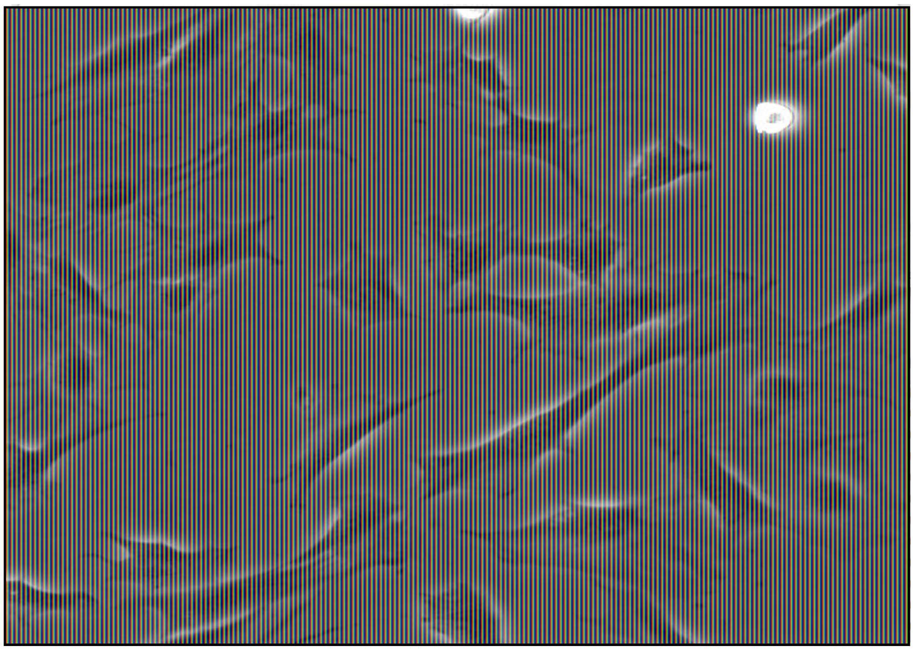

Supplementary Movie S1: Related to Figure 1: Representative video of WT cells followed during 12 hours. It allowed determination of cells speed and directionality.

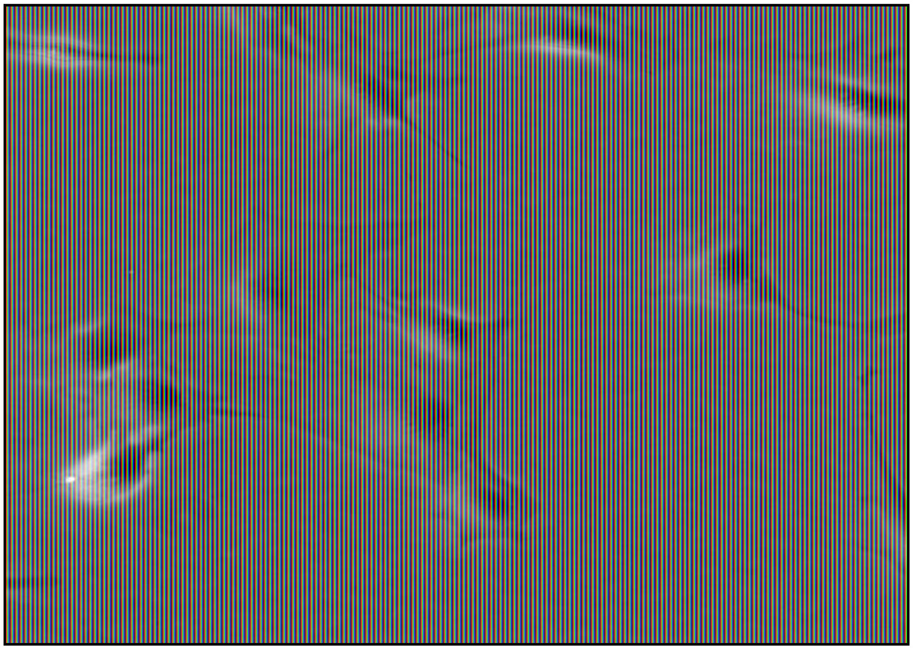

Supplementary Movie S2: Related to Figure 1: Representative video of Sdhb−/− cells followed during 12 hours. It allowed determination of cells speed and directionality.
